# Endoscopic Vacuum Therapy in Patients with Transmural Defects of the Upper Gastrointestinal Tract: A Systematic Review with Meta-Analysis

**DOI:** 10.3390/jcm10112346

**Published:** 2021-05-27

**Authors:** Da Hyun Jung, Hae-Ryong Yun, Se Joon Lee, Na Won Kim, Cheal Wung Huh

**Affiliations:** 1 Department of Internal Medicine, Severance Hospital, Yonsei University College of Medicine, Seoul 03722, Korea; JUNGDH@yuhs.ac; 2 Department of Internal Medicine, Yongin Severance Hospital, Yonsei University College of Medicine, Seoul 03722, Korea; siberian82@yuhs.ac (H.-R.Y.); LEESJ@yuhs.ac (S.J.L.); 3 Medical Library, Yonsei University College of Medicine, Seoul 03722, Korea; NWKIM@yuhs.ac

**Keywords:** endoscopic vacuum therapy, etiology, transmural defect, upper gastrointestinal tract

## Abstract

A transmural defect of the upper gastrointestinal (UGI) tract is a life-threatening condition associated with high morbidity and mortality. Recently, endoscopic vacuum therapy (EVT) was used for managing UGI defects and showed promising results. We conducted a systematic review and meta-analysis to synthesize evidence on the efficacy of EVT in patients with transmural defects of the UGI tract. We searched the PubMed, Cochrane Library, and Embase databases for publications on the effect of EVT on successful closure, mortality, complications, and post-EVT strictures. Methodological quality was assessed using the Newcastle–Ottawa quality assessment scale. This meta-analysis included 29 studies involving 498 participants. The pooled estimate rate of successful closure with EVT was 0.85 (95% confidence interval [CI]: 0.81–0.88). The pooled estimate rates for mortality, complications, and post-EVT strictures were 0.11, 0.10, and 0.14, respectively. According to the etiology of the transmural defect (perforation vs. leak and fistula), no significant difference was observed in successful closure (odds ratio [OR]: 1.45, 95% CI: 0.45–4.67, *p* = 0.53), mortality (OR: 0.77, 95% CI: 0.24–2.46, *p* = 0.66), complications (OR: 0.94, 95% CI: 0.17–5.15, *p* = 0.94), or post-EVT stricture rates (OR: 0.70, 95% CI: 0.12–4.24, *p* = 0.70). The successful closure rate was significantly higher with EVT than with self-expanding metal stent (SEMS) placement (OR: 3.14, 95% CI: 1.23–7.98, *p* = 0.02). EVT is an effective and safe treatment for leaks and fistulae, as well as for perforations in the UGI. Moreover, EVT seems to be a better treatment option than SEMS placement for UGI defects.

## 1. Introduction

Transmural defects of the upper gastrointestinal (UGI) tract are categorized as perforations, leaks, or fistulae. A perforation is defined as an acute rupture of the gastrointestinal wall that can occur after an endoscopic procedure or due to underlying pathology, such as massive vomiting (Boerhaave syndrome), foreign bodies, peptic ulcers [1,2]. A leak is a communication between the intraluminal and extraluminal spaces, which occurs because of postsurgical complications, most commonly at the anastomosis site. A fistula that develops owing to prolonged anastomotic leak is defined as an abnormal connection between the gastrointestinal tract and other organs or abscess cavities. Tracheoesophageal fistula is representative. Transmural defects of the UGI are life-threatening and associated with high morbidity and mortality rates [3,4]. The optimal management of UGI transmural defects remains controversial. Though surgery is an important treatment strategy, the associated mortality rate is about 12–50% [3,5,6]. Placement of a self-expanding metal stent (SEMS) was also proven to be an effective treatment strategy for UGI defects [7,8]. However, SEMS placement can also cause complications such as stent migration, stent ingrowth, perforation, bleeding, epidural abscess, and vascular fistula [9,10,11].

Recently, endoscopic vacuum therapy (EVT) was used with promising results for managing UGI defects [12,13,14]. This method involves the application of a continuous negative pressure to drain the infected fluid and accelerates wound healing [15]. EVT is suitable for localized defects for which stent placement is not feasible. Moreover, external drainage is not necessary in most cases [16]; however, the clinical success rate of EVT varies widely from 66.7–100% [17,18,19]. In addition, corroborating evidence is needed because most studies are limited to case series and retrospective cohort studies with small sample sizes.

We performed a meta-analysis of studies on the clinical outcomes of EVT in patients with transmural defects of the UGI tract. We aimed to assess the effect of EVT on successful closure, mortality, postprocedural complications, and stricture. In addition, we evaluated the efficacy of EVT according to the etiology of the transmural defect (perforation vs. leak and fistula) and treatment method (EVT vs. SEMS placement).

## 2. Material and Methods

### 2.1. Literature Search Strategy

We performed a systematic review and meta-analysis following the principles of the PRISMA (Preferred Reporting Items for Systematic reviews and Meta-Analyses) statement [20]. The PubMed, Cochrane Library, and Embase databases (from inception to April 2020) were independently searched by three authors (DHJ, HRY, and CWH). We used the following search string: anastomotic leak OR anastomotic leakage OR postoperative leak OR postoperative leakage OR esophageal leak OR esophageal leakage OR esophageal fistula OR leakage OR fistula OR leak OR perforation OR upper gastrointestinal tract OR esophagus OR esophageal OR gastric OR stomach OR esophagectomy OR anastomosis AND endoscopic vacuum therapy OR endoscopic vacuum-assisted closure OR endoluminal vacuum therapy OR vacuum therapy OR vacuum-assisted closure OR negative pressure wound therapy OR endoscopic negative pressure therapy OR negative pressure therapy OR endovac therapy OR endo sponge (as illustrated in supplementary Table S1). We manually and repetitively searched the cited references in published studies to identify other studies.

### 2.2. Study Selection

In the first stage of the study selection, the titles and abstracts of the articles that our keyword search returned were scrutinised to rule out irrelevant articles. Thereafter, the full texts of all selected studies were screened according to our inclusion and exclusion criteria. The inclusion criteria were as follows: (1) a diagnosis of perforation, leak, or fistula of the UGI tract; (2) EVT as a primary or rescue treatment; and (3) investigations of adults aged ≥18 years. The exclusion criteria were as follows: (1) article types other than original articles; (2) case reports including fewer than two patients; (3) abstract-only publications; and (4) publications in a language other than English. Only the most recent study was selected if several publications covering the same study population existed.

### 2.3. Data Extraction

Three authors (DHJ, HRY, and CWH) of this review independently extracted data from the included studies using a predata extraction form. Further, we reviewed the titles and abstracts of all the included studies to exclude irrelevant publications. Any discrepancies in data interpretation were resolved through discussions, rereview of studies, and consultation with another author (SJL). We extracted the following information: year of publication, first author, study design, patient age and sex, sample size, study region, follow-up duration, transmural defect size, time to diagnosis, time to treatment, EVT type, successful closure rate, mortality rate, complication rate, post-EVT stricture rate, hospital length of stay, intensive care unit length of stay, treatment duration, and number of sponge or stent changes.

### 2.4. Primary and Secondary Outcomes

The primary outcome was the successful closure rate. Successful closure was defined as no evidence of leakage on direct endoscopic visualization and the absence of contrast extravasation on either a computed tomography scan with oral contrast, esophagography, or a UGI study. The secondary outcomes were mortality rate, complication rate (Clavien–Dindo score ≥ 3), and stricture rate after EVT.

### 2.5. Methodological Quality

The Newcastle–Ottawa quality assessment scale for cohort studies was used to evaluate the risk of bias. This scale rates studies on three sources of bias (selection, comparability, and outcome) based on eight criteria. Each criterion is rated with 1 star except comparability, which is rated a maximum of 2 stars. For this systematic review, the studies scoring 7–9 stars were defined be of low risk of bias, the studies scoring 4–6 stars were defined to be of moderate risk of bias, and the studies scoring 1–3 stars were defined to be of high risk of bias. Three authors (CWH, HRY, and DHJ) independently evaluated the methodological quality of the selected studies. Any disagreement between the three authors was resolved through discussions.

### 2.6. Statistical Analysis

A meta-analysis was performed using the statistical software R (version 3.3.3; R Foundation for Statistical Computing, Vienna, Austria). The Mantel–Haenszel random-effect model was applied to binary endpoints. The random-effects model was selected because it considers the possibility of heterogeneity. The median difference was used for continuous variables. Pooled medians were estimated using the quantile estimation method. In addition, we performed subgroup analyses according to the following criteria: closure rate, mortality rate, complication rate, post-EVT stricture rate according to the etiology of transmural defect (perforation vs. leak and fistula), closure rate, mortality, treatment duration, hospital stay, and number of sponge/stent changes of EVT and SEMS.

The *I*^2^ test developed by Higgins was used to determine heterogeneity [21]. This test measures the percentage of total variation across studies. In cases of significant heterogeneity (*I*^2^ > 25%), the methodological section of each publication was re-evaluated to determine whether any discrepancy could be checked. We used the Egger test to assess the extent of the publication bias. Statistical significance was set at *p* < 0.05.

## 3. Results

### 3.1. Study Selection

**Figure 1:**
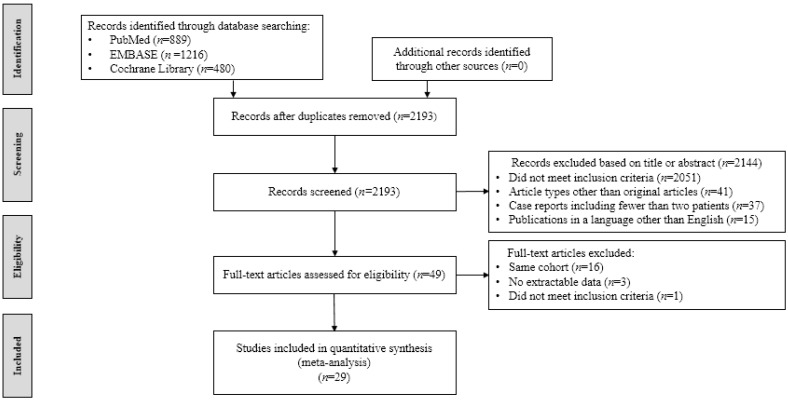
Flowchart of studies included in meta-analysis.

A total of 2585 studies were identified. Duplicate articles (*n* = 392) were excluded. Further, 2144 articles were rejected based on the title and abstracts. Forty-nine articles were reviewed. After assessing eligibility, 20 articles were excluded (as illustrated in Figure 1). Finally, a total of 29 articles were included involving 498 participants [13,14,18,22,23,24,25,26,27,28,29,30,31,32,33,34,35,36,37,38,39,40,41,42,43,44,45,46,47]

### 3.2. Study Characteristics and Methodological Quality

The baseline characteristics of the included studies are presented in Table 1. Nineteen articles were retrospective cohort studies, and 10 were case series. Eight studies included only patients with postoperative leaks, [13,23,25,26,33,42,43,46] and two studies included only patients with perforations [24,27]. Eleven studies included patients with both postoperative leaks and perforations [14,18,29,31,32,34,35,38,40,41,44]. Four studies included patients with a fistula [36,37,45,47]. Four studies compared EVT with SEMS placement [22,28,30,39]. A total of 24 studies were conducted in Western countries (Germany 14, United States 4, Switzerland 2, United Kingdom 2, Portugal 1, and Australia 1), whereas five studies were conducted in Asia (Korea 4 and China 1).

Table 2 summarizes the clinical outcomes of the included studies. All studies except two [23,36] reported the successful closure rate. Mortality was reported in all studies except one [40]. The complications and post-EVT stricture rates were reported in 21 and 16 studies, respectively. Hospital stay, intensive care unit stay, duration of therapy, and the number of sponge changes were reported in 14, 5, 23, and 22 studies, respectively.

The definition of clinical success, detailed indications of treatment, and causes of mortality in the included studies are shown in supplementary Table S2. In addition, four studies that included fistula cases are summarized in supplementary Table S3.

The patient characteristics of studies comparing EVT and SEMS placement are summarized in supplementary Table S4. Brangewitz et al. [22] reported successful closure, mortality, duration of treatment, length of hospital stay, and stricture development in 71 patients with leaks or perforations after esophagectomies, fundoplications, esophageal diverticulotomies, Boerhaave syndrome, and iatrogenic perforations, and compared EVT (n = 32) with SEMS placement (*n* = 39). Schniewind et al. [23] assessed 47 patients diagnosed with postoperative leaks after esophagectomy. Mortality and length of hospital stay were compared between patients treated with EVT (*n* = 17) and SEMS placement (*n* = 12). Mennigen et al. [28] showed that successful closure, mortality, duration of treatment, length of hospital stay, and adverse events were analysed in 45 patients who were diagnosed with postoperative leak following esophagectomy in comparisons between EVT (*n* = 15) versus SEMS (*n* = 30). Hwang et al. [30] compared EVT (*n* = 7) and SEMS placement (*n* = 11) in South Korea. Although the number of enrolled patients was small, they also showed successful closure, duration of treatment, length of hospital stay, and adverse events. They included eighteen patients who were diagnosed with postoperative leak after esophagectomy or gastrectomy for cancer treatment. Lastly, Berlth et al. [39] reported successful closure, mortality, duration of treatment, length of hospital stay, and adverse events in comparisons between EVT (*n* = 34) and SEMS (*n* = 77). One hundred and eleven patients underwent curative surgery to treat malignancies and were diagnosed with postoperative leaks.

The methodological quality of the studies is presented in Supplementary Table S5. The quality was poor in 15 studies [24,25,27,29,32,34,36,37,38,41,42,43,44,45,47] and moderate in 14 studies [13,14,18,22,23,26,28,30,31,33,35,39,40,46].

### 3.3. Primary and Secondary Outcomes

#### 3.3.1. Primary Outcome—Successful Closure Rate

**Figure 2:**
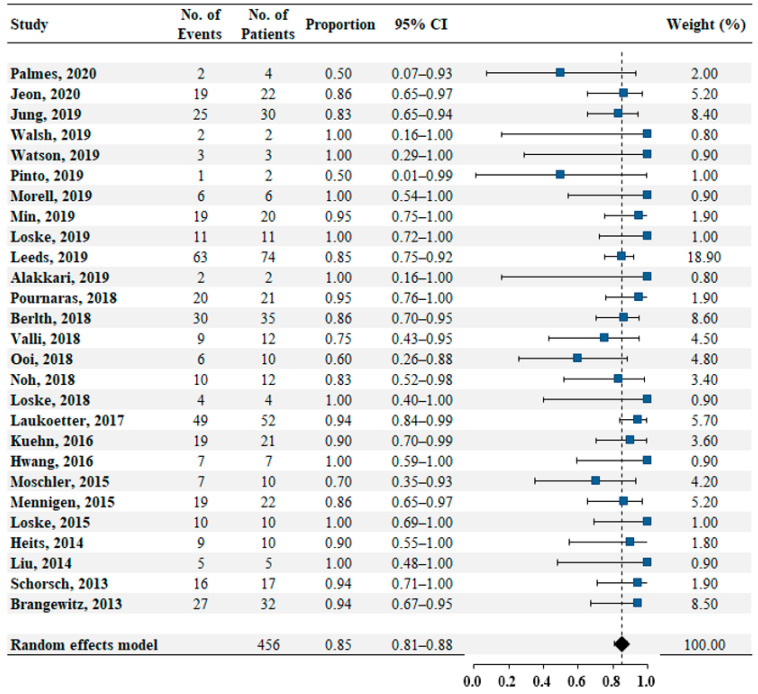
Pooled estimate rate for successful closure in patients with transmural defects of upper gastrointestinal tract.

Twenty-seven studies reported data on successful closure in 456 patients. The pooled estimate rate for successful closure was 0.85 (95% confidence interval [CI]: 0.81–0.88, Figure 2). No heterogeneity was found among the studies (*I*^2^ = 0%, *p* = 0.68). No publication bias was detected by the Egger test (*p* = 0.33).

#### 3.3.2. Secondary Outcomes—Mortality, Complication, and Post-EVT Stricture Rates

**Figure 3:**
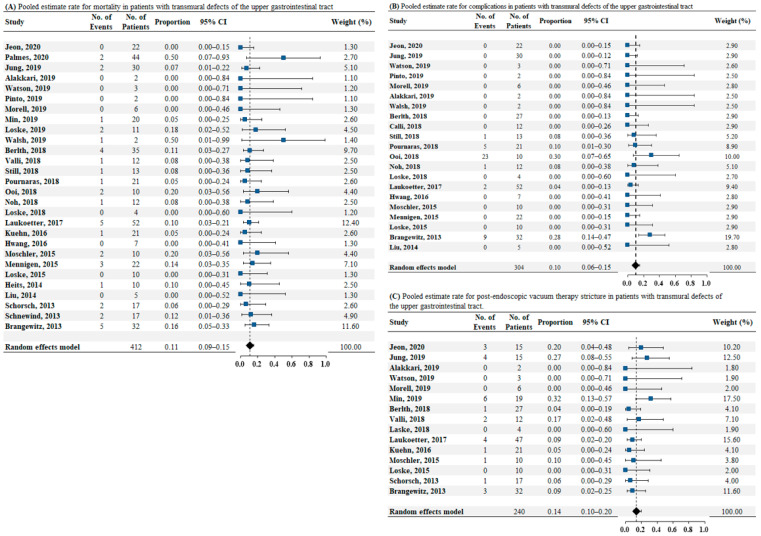
(**A**) Pooled estimate rate for mortality in patients with transmural defects of upper gastrointestinal tract. (**B**) Pooled estimate rate for complications in patients with transmural defects of the upper gastrointestinal tract. (**C**) Pooled estimate rate for postendoscopic vacuum therapy stricture in patients with transmural defects of upper gastrointestinal tract. Abbreviations: No, number; OR, odds ratio; CI, confidence interval.

Data on mortality were reported in 28 studies comprising a total of 412 patients. The pooled estimated mortality rate was 0.11 (95% CI: 0.09–0.15, as illustrated in Figure 3A). No heterogeneity was found among these studies (*I*^2^ = 0%, *p* = 0.96). No publication bias was detected by the Egger test (*p* = 0.38). Twenty-one studies reported data on complications in 304 patients. The pooled estimate rate for complications was 0.10 (95% CI: 0.06–0.15, as illustrated in Figure 3B). Low heterogeneity was found among the studies (*I*^2^ = 13.8%, *p* = 0.28). Publication bias was detected by the Egger test (*p* < 0.05). Sixteen studies reported data on post-EVT strictures in 240 patients. The pooled estimate rate for post-EVT stricture was 0.14 (95% CI: 0.10–0.20, as illustrated in Figure 3C). No heterogeneity was found among these studies (*I*^2^ = 0%, *p* = 0.45). The *p*-value of publication bias by the Egger test was 0.06.

### 3.4. Subgroup Analysis

#### 3.4.1. Perforation vs. Leak and Fistula—Successful Closure, Mortality, Complications, and Post-EVT Stricture Rates

**Figure 4:**
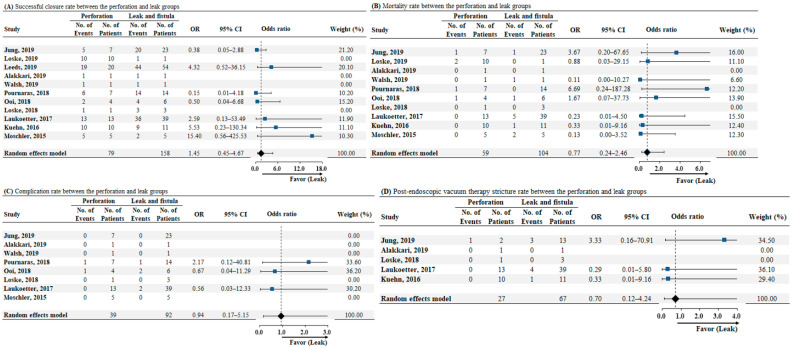
(**A**) Forrest plot of successful closure rate for comparison between the perforation and leak group. (**B**) Forrest plot of mortality rate for comparison between perforation and leak groups. (**C**) Forrest plot of complication rate for comparison between perforation and leak groups. (**D**) Forrest plot of postendoscopic vacuum therapy stricture rate for comparison between perforation and leak groups. Abbreviations: No, number; OR, odds ratio; CI, confidence interval.

According to the etiology of the transmural defect, evaluation of the successful closure rate was performed in 11 studies. The pooled analysis showed that the successful closure rate was similar between the perforation and leak groups (odds ratio [OR]: 1.45, 95% CI: 0.45–4.67, *p* = 0.53; as illustrated in Figure 4A). We detected low heterogeneity among the studies (*I*^2^ = 24.1%, *p* = 0.24). Data on mortality according to the etiology of transmural defects were available for 10 studies. The analysis revealed no significant difference between the two groups in terms of mortality rate (OR: 0.77, 95% CI: 0.24–2.46, *p* = 0.66; as illustrated in Figure 4B), and there was no heterogeneity (*I*^2^ = 0%, *p* = 0.58). Eight studies reported data on complications according to the etiology of transmural defects. The pooled analysis showed that the complication rates were similar between the perforation and leak groups (OR: 0.94, 95% CI: 0.17–5.15, *p* = 0.94; as illustrated in Figure 4C). No heterogeneity was detected among the studies (*I*^2^ = 0%, *p* = 0.79). Data on post-EVT stricture rate according to the etiology of transmural defects were available for five studies. No significant difference was observed between the two groups in terms of post-EVT stricture rate (OR: 0.70, 95% CI: 0.12–4.24, *p* = 0.70; as illustrated in Figure 4D), and no heterogeneity was noted (*I*^2^ = 0%, *p* = 0.47).

#### 3.4.2. EVT vs. SEMS—Successful Closure, Mortality, Treatment Duration, Length of Hospital Stay, and the Number of Endoscopic Stent/Sponge Changes

**Figure 5:**
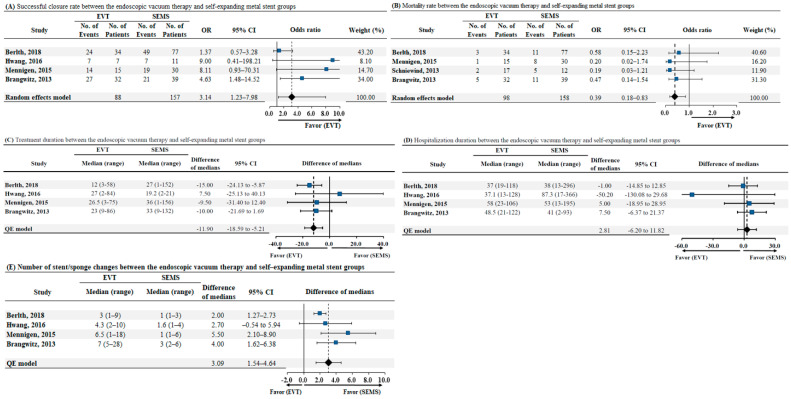
(**A**) Successful closure rate between endoscopic vacuum therapy and self-expanding metal stent groups, (**B**) Mortality rate between endoscopic vacuum therapy and self-expanding metal stent groups, (**C**) Treatment duration between endoscopic vacuum therapy and self-expanding metal stent groups, (**D**) Hospitalization duration between endoscopic vacuum therapy and self-expanding metal stent groups, (**E**) Number of stent/sponge changes between endoscopic vacuum therapy and self-expanding metal stent groups. Abbreviations: EVT, endoscopic vacuum therapy; SEMS self-expanding metal stent; No, number; OR, odds ratio; CI, confidence interval; QE, quantitative estimation.

The length of hospital stay was mentioned in all included studies. Among the four studies that compared EVT and SEMS placement, successful closure rate, mortality rate, duration of treatment, and the number of endoscopic stent/sponge changes were demonstrated. The successful closure rate was significantly higher in the EVT group than in the SEMS group (OR: 3.14, 95% CI: 1.23–7.98, *p* = 0.02) (as illustrated in Figure 5A). The mortality rate was lower in the EVT group than in the SEMS group (OR: 0.39, 95% CI: 0.18–0.83, *p* = 0.01) (as illustrated in Figure 5B). Compared to SEMS placement, EVT showed a shorter treatment duration, with an estimated pooled median difference of 11.90 days (95% CI: −18.59–−5.21, *p* < 0.01), after excluding one study that reported a shorter duration of treatment with SEMS placement (as illustrated in Figure 5C). The length of hospital stay showed similar results between the EVT and SEMS groups with an estimated pooled median difference of 2.81 days (95% CI: 6.20–11.82, *p* = 0.27) (as illustrated in Figure 5D). In addition, the number of endoscopic stent/sponge changes were significantly higher in EVT than with SEMS placement, and an estimated pooled median difference of 3.09 was noted (95% CI 1.54–4.64, *p* = 0.03)) (as illustrated in Figure 5E).

## 4. Discussion

To date, many studies reported promising outcomes in patients with transmural defects of the UGI tract with EVT used as a definitive treatment. However, these previous studies included only a limited number of patients. Recently, several systematic reviews reported the usefulness of EVT in transmural defects of the UGI tract. [19,48,49,50]; however, these reviews were only descriptive and did not conduct statistical analysis with a summary estimate. Therefore, a meta-analysis is needed to compile and analyze the available data on the efficacy of EVT in transmural defects of the UGI tract. Our meta-analysis included case series in which a single group was assessed with no intrastudy comparisons. Nevertheless, this meta-analysis has an advantage over narrative reviews because it assessed effect sizes and integrated them into a single statistical analysis.

In this meta-analysis, the closure rate of transmural UGI defects with EVT was excellent (85%), and EVT was associated with low mortality (11%), complications (10%), and post-EVT stricture rates (14%) rates. Moreover, no significant difference was observed in successful closure (OR: 1.45, 95% CI: 0.45–4.67), mortality (OR: 0.77, 95% CI: 0.24–2.46), complications (OR: 0.94, 95% CI: 0.17–5.15, *p* = 0.94), and post-EVT stricture rates (OR: 0.70, 95% CI: 0.12–4.24, *p* = 0.70) according to the etiology of the transmural defect (perforation vs. leak and fistula). Although the etiology of transmural UGI defects was different, the efficacy of EVT was similar between the groups.

EVT had a significantly higher successful closure rate than with SEMS placement (OR: 3.14, 95% CI: 1.23–7.98). In addition, the mortality rate was lower (OR: 0.39, 95% CI: 0.18–0.83) and the treatment duration was shorter with EVT than with SEMS placement (−11.90, 95% CI: −18.59–−5.21). We believe that this was due to the difference in methodology between EVT and SEMS placement. Generally, SEMS removal or replacement is performed 4–6 weeks after SEMS insertion. Therefore, the successful closure rate with SEMS treatment was determined 4–6 weeks after the previous SEMS insertion. In contrast, because EVT is repeated every 3–5 days, clinicians can also check successful closure every 3–5 days. Therefore, successful closure could be detected sooner with EVT than with SEMS placement. In addition, EVT treatment could offer the possibility of performing endoscopic lavage and debridement with every change, which was shown to reduce pleural inflammation and leakage-associated mortality.

The principle of EVT is similar to the classical vacuum-assisted closure treatment, which is a well-established therapy for chronic superficial wounds [51]. In EVT, a polyurethane sponge is placed inside the defect to apply negative pressure. Defect healing is achieved through continuous abscess drainage, thus decreasing bacterial colonization, enhancing vascularity, and promoting tissue granulation [51,52]. An internal vacuum sponge (endo-SPONGE) device was first successfully used for treating a UGI anastomosis leak in 2008 [15]. Since then, EVT was used to manage UGI defects and showed good short- and long-term clinical outcomes. SEMS placement also showed effective outcomes for UGI defects [7,8]. However, stent therapy is usually accompanied by additional abscess drainage, local pressure necrosis of the mucosa, stent migration, stent ingrowth, bleeding, and perforation. Surgery is also one of the strategies for treating transmural defects of the UGI; however, it is associated with a high mortality rate [5,6]. To date, comparative studies assessing different treatment modalities for UGI defects are rare [53]. Therefore, clinical evidence of efficacy of EVT for treatment of UGI defects is still inadequate for directing treatment modalities. Our meta-analysis showed that EVT is an effective and safe treatment method for treating leaks, fistulae, and perforations.

Usually, transmural defects of the UGI tract are classified as perforations, leaks, or fistulae. Of these, fistulae are the most difficult to close because the epithelial tract is often fibrotic, and these arise in unhealthy tissues, which are inflamed, damaged, or ischemic. Although the included cases were too few (n = 8), this meta-analysis showed a successful closure rate of 50% in patients with a fistula. Given the inadequate response of fistulae to other treatments such as SEMS placement, EVT is a promising option for treating patients with fistula.

The major disadvantages of EVT are the need for repetitive endoscopic procedures, nasogastric tube-related discomfort, and sponge dislocation. The main and most dreadful event associated with EVT is massive bleeding [19,48]. It can occur from a fistula between the cavity and main vessels and from rupture of a pseudoaneurysm from circumjacent vessels or heart chambers. More frequent changes of the sponge may help prevent or reduce the risk of severe bleeding. Moreover, massive bleeding can occur in cases of intracavitary therapy in which direct contact with blood vessels is possible. Therefore, intraluminal EVT may be safer than intracavitary EVT. Additionally, computed tomography scans should be reviewed before initiating intracavitary EVT to exclude vascular complications. In our review, post-EVT strictures occurred in 14% of cases; however, all strictures were easily resolved through endoscopic dilatations (26 cases).

Although the results of this study are promising, it had several limitations. All included studies were retrospective in nature without randomisation. This could have resulted in a selection bias in this study. Typically, the choice of modalities (EVT, SEMS placement, operation, and nonoperative management) were chosen according to the severity of the patients. Patients managed conservatively tend not to be septic and have a contained leak versus those who have apparent mediastinal contamination and warrant endoscopic or surgical intervention. As EVT is a relatively new treatment method, it could be assumed that the first experience of the studies included in this meta-analysis was performed in cases in which a favorable outcome was expected, thus influencing the results. Although randomized controlled trials are considered the best method for evaluating treatment effects, performing such trials would be difficult owing to ethical concerns and methodological difficulties. Second, although the statistical heterogeneity was low, the clinical heterogeneity was high among the included studies. Patient heterogeneity and detailed indication were different among the included studies. Therefore, the complexity and comorbidities of each patient could affect the treatment success. In addition, SEMS placement is a more standard treatment compared to EVT, which may also affect treatment outcomes. To address this limitation, we have additionally summarized detailed information of the studies included in this meta-analysis (as illustrated in supplementary Tables S1–S4) Third, the included studies had a limited quality. Fourth, the sample size of each study was insufficient to reach definitive conclusions. Therefore, additional data are needed to define the role of EVT in patients with UGI defects. Finally, most of the included studies were from Western countries, especially Germany. Large-scale studies from other regions are required to validate the usefulness of EVT in treating UGI defects in patients of different ethnicities. Despite these limitations, to the best our knowledge, this meta-analysis contains the most comprehensive analysis of the effectiveness of EVT for treating UGI defects.

## 5. Conclusions

In summary, this meta-analysis revealed that EVT could be an effective and safe treatment method for leaks and fistulae as well as perforations in the UGI. In addition, EVT may be a better treatment option than SEMS placement for UGI defects. However, a definite recommendation cannot be made for the treatment of UGI defects due to the limitations of the included studies mentioned above. We believe that prospective large-scale studies from various regions worldwide are needed to validate the effectiveness of EVT for treating UGI defects.

## Figures and Tables

**Table 1:** Characteristics of 29 studies included.

Authors	Study Design	*N*	Male (n)	Age (Median, Years)	BMI (Median)	Region of Study	Follow-Up (Median, Months)	Method of Diagnosis	Defect Size (Median, mm)	Time to Diagnosis (Median, Days)	Time to Treatment (Median, Days)	Intracavitary/Intraluminary
Palmes 2020	Case series	Fistula: 4	NA	NA	NA	Western (Germany)	MA	Endoscopy CT Esophagogram	NA	NA	NA	NA
Jung 2019	Retrospective	Leak: 23 Perforation: 7	20	65.1 ^†^	NA	Western (Germany)	11.8 ^†^	NA	NA	Leak: 8.5 Perforation: <1	NA	IC: 6, IL: 24
Jeon 2019	Retrospective	Leak: 22	17	68	NA	Eastern (Korea)	29.8	Endoscopy CT Esophagogram	NA	11	NA	IC: 10, IL: 12
Watson 2019	Case series	Leak: 2 Fistula: 1	2	69.6 ^†^	NA	Western (USA)	2.7	Endoscopy	NA	NA	NA	NA
Pinto 2019	Case series	Leak: 2	1	44.0 ^†^	NA	Western (Portugal)	NA	Endoscopy CT	NA	NA	NA	IC: 2, IL: 1
Morell 2019	Case series	Leak: 6	2	49.0 ^†^	44.2	Western (Switzerland)	NA	Endoscopy CT	20^†^	4.5 ^†^	3.3 ^†^	NA
Min 2019	Retrospective	Leak: 20	20	66.5	NA	Eastern (Korea)	7.1	Endoscopy Esophagogram	17.5	12.5	3	IC: 20
Loske 2019	Case series	Leak: 1 Perforation: 10	4	65.7 ^†^	NA	Western (Germany)	NA	Endoscopy CT	NA	NA	NA	IC: 1 IL: 10
Leeds 2019	Retrospective	Leak: 54 Perforation: 20	NA	NA	NA	Western (USA)	NA	NA	NA	NA	NA	NA
Walsh 2019	Case series	Leak: 1 Perforation: 1	1	69.5	NA	Western (USA)	NA	NA	20	21	NA	NA
Alakkari 2019	Case series	Leak: 1 Perforation: 1	0	67 ^†^	NA	Western (UK)	NA	NA	NA	NA	19 ^†^	IC: 2
Berlth 2018	Retrospective	Leak: 111 EVT: 34 SEMS 77	92	EVT: 65 SEMS: 64	EVT: 26 SEMS: 26	Western (Germany)	NA	Endoscopy CT Esophagogram	NA	EVT: 8 SEMS: 8	EVT: 0 SEMS: 0	NA
Valli 2018	Retrospective	Leak: 11 Fistula: 1	9	Leak: 57.5 Fistula: 80	NA	Western (Switzerland)	NA	Endoscopy CT Esophagogram	NA	NA	Leak: 18.4 Fistula: 52	IC: 7, IL: 5
Still 2018	Retrospective	Leak: 2 Perforation: 9 Fistula: 2	6	63	23	Western (USA)	NA	Endoscopy CT Esophagogram	NA	NA	NA	IC: 3, IL: 7
Pournaras 2018	Retrospective	Leak: 14 Perforation: 7	NA	NA	NA	Western (UK)	NA	NA	NA	NA	NA	NA
Ooi 2018	Retrospective	Leak: 6 Perforation: 4	NA	56.7 ^†^	NA	Western (Australia)	NA	NA	18.3	NA	33.6	NA
Noh 2018	Retrospective	Leak: 12	12	57.0	NA	Eastern (Korea)	12.9	CT Esophagogram	13	13.5	11	IC: 3, IL: 9
Loske 2018	Case series	Leak: 3 Perforation: 1	NA	NA	NA	Western (Germany)	NA	NA	NA	NA	NA	NA
Laukoetter 2017	Retrospective	Leak: 39 Perforation: 13	31	65	NA	Western (Germany)	5.4	Endoscopy CT Esophagogram	NA	NA	8	NA
Kuehn 2016	Retrospective	Leak: 11 Perforation: 10	15	72	NA	Western (Germany)	17	Endoscopy CT Esophagogram	NA	NA	NA	IC: 10, IL: 11
Hwang 2016	Retrospective	Leak: 18 EVT: 7 SEMS 11	14	EVT: 71.1 SEMS: 67.3	NA	Eastern (Korea)	NA	NA	EVT: 8.1 SEMS: 6.6	NA	NA	NA
Möschler 2015	Retrospective	Leak: 5 Perforation: 5	5	73.9 ^†^	NA	Western (Germany)	4	NA	NA	NA	NA	IC: 6, IL: 4
Mennigen 2015	Retrospective	Leak: 45 EVT: 15 SEMS 30	35	EVT: 56 SEMS: 65.5	NA	Western (Germany)	EVT: 8.3 SEMS: 16.8	Endoscopy CT Esophagogram	NA	EVT: 7 SEMS: 7	NA	IC: 22
Loske 2015	Case series	Perforation: 10	NA	NA	NA	Western (Germany)	2.8	Endoscopy	19	<1	NA	IC: 1, IL: 9
Heits 2014	Retrospective	Perforation: 10	5	66 ^†^	NA	Western (Germany)	9	Endoscopy CT Esophagogram	4.2	NA	NA	NA
Liu 2014	Case series	Leak: 5	NA	61.8 ^†^	NA	Eastern (China)	NA	Endoscopy CT Esophagogram	NA	9.2	NA	NA
Schorsch 2013	Retrospective	Leak: 17	NA	NA	NA	Western (Germany)	NA	NA	14.7	10	NA	IC: 8, IL: 9
Schniewind 2013	Retrospective	Leak: 47	NA	NA	NA	Western (Germany)	NA	NA	NA	NA	NA	NA
Brangewitz 2013	Retrospective	EVT:32 SEMS: 39	58	EVT:63 SEMS: 62	EVT: 25.2 SEMS: 26.4	Western (Germany)	NA	NA	NA	NA	NA	NA

EVT, endoscopic vacuum therapy; CT, computed tomography; IC, intracavitary; IL, intraluminary; NA, not available; SEMS, self-expanding metal stent ^†^ Data expressed as mean.

**Table 2:** Clinical outcomes of 29 studies included.

Authors	N	Successful Closure Rate (*n*, %)	Mortality Rate (*n*, %)	Complication Rate (*n*, %)	Stricture Rate (*n*, %)	Hospital Stay (Median, Days)	ICU Stay (Median, Days)	Duration of Therapy (Median, Days)	Sponge Changes
Palmes 2020	Fistula: 4	2/4 (50)	2/4 (50)	NA	NA	NA	NA	88.5	NA
Jung 2019	Leak: 23	20/23 (87.0)	1/23 (4.3)	0/23 (0)	3/13 (23.1)	54.4	NA	15.7	3.4
	Perforation: 7	5/7 (71.4)	1/7 (14.3)	0/7 (0)	1/2 (50.0)	33.7		27.0	6.4
Jeon 2019	Leak: 22	19/22 (86.4)	0/22 (0)	0/22 (0)	3/15 (20.0)	24	NA	13	3
Watson 2019	Leak: 2	2/2 (100.0)	0/2 (0)	0/2 (0)	0/2 (0)	NA	NA	16	3
	Fistula: 1	1/1 (100.0)	0/1 (0)	0/1 (0)	0/1 (0)			40	9
Pinto 2019	Leak: 2	1/2 (50.0)	0/2 (0)	0/2 (0)	NA	NA	NA	22	3
Morell 2019	Leak: 6	6/6 (100.0)	0/6 (0)	0/6 (0)	0/6 (0)	39.8 ^†^	10.2 ^†^	32.3 ^†^	4
Min 2019	Leak: 20	19/20	1/20	NA	6/19	49	NA	14.5	5
Loske 2019	Leak: 1	1/1 (100.0)	0/1 (0)	NA	NA	NA	NA	11	1.8
	Perforation: 10	10/10 (100.0)	2/10 (20.0)						
Leeds 2019	Leak: 54	44/54 (81.5)	NA	NA	NA	NA	NA	NA	NA
	Perforation: 20	19/20 (95.0)							
Walsh 2019	Leak: 1	2/2 (100.0)	1/1 (100.0)	0/1 (0)	NA	NA	NA	55	10
	Perforation: 1		0/1 (0)	0/1 (0)				42	3
Alakkari 2019	Leak: 1	1/1 (100.0)	0/1 (0)	0/1 (0)	0/1 (0)	NA	NA	28	6
	Perforation: 1	1/1 (100.0)	0/1 (0)	0/1 (0)	0/1 (0)			56	13
Berlth 2018	Leak: 111 EVT: 34 SEMS 77	24/34 (70.6)	3/34 (0.9)	0/27 (0)	1/27 (3.7)	37	8	12	3
		49/77 (63.6)	11/77 (14.3)	13/69 (18.8)	5/69 (7.2)	38	7	27	1
Valli 2018	Leak: 11	9/11 (81.8)	0/11 (0)	0/11 (0)	2/11 (18.1)	NA	NA	20.8	5
	Fistula: 1	0/1 (0)	1/1 (100.0)	0/1 (0)	0/1 (0)			16	4
Still 2018	Leak: 2	NA	1/13 ^‡^	1/13 ^‡^	NA	NA	NA	NA	NA
	Perforation: 9								
	Fistula: 2								
Pournaras 2018	Leak: 14	14/14 (100.0)	0/14 (0)	1/14 (7.1)	NA	35	NA	NA	7
	Perforation: 7	6/7 (85.7)	1/7 (14.3)	1/7 (14.3)					
Ooi 2018	Leak: 6	4/6 (66.7)	1/6 (16.7)	2/6 (33.3)	NA	62	12	25.5 ^†^	8.3 ^†^
	Perforation: 4	2/4 (50.0)	1/4 (25.0)	1/4 (25.0)					
Noh 2018	Leak: 12	10/12 (83.3)	1/12 (8.3)	1/12 (8.3)	1/12 (8.3)	NA	NA	25	2.7 ^†^
Loske 2018	Leak: 3	3/3 (100.0)	0/3 (0)	0/3 (0)	0/3 (0)	NA	NA	NA	NA
	Perforation: 1	1/1 (100.0)	0/1 (0)	0/1 (0)	0/1 (0)				
Laukoetter 2017	Leak: 39	36/39 (92.3)	5/39 (12.8)	2/39 (5.1) ^§^	4/39 (10.2)	60	NA	20	6
	Perforation: 13	13/13 (100.0)	0/13 (0)	0/13 (0)	0/13 (0)	46		24	6
Kuehn 2016	Leak: 11	9/11 (81.8)	1/11 (18.2)	NA	1/11 (18.2)	NA	NA	12	4
	Perforation: 10	10/10 (100.0)	0/10 (0)		0/10 (0)			15	5
Hwang 2016	Leak: 18 EVT: 7 SEMS 11	7/7 (100.0)	NA	0/7 (0)	NA	37.1	NA	27	4.3
		7/11 (63.6)		6/11 (54.5)		87.3		19.2	1.6
Möschler 2015	Leak: 5	2/5 (40.0)	2/5 (40.0)	0/5 (0)	1/10 (10.0) ^§^	38	NA	34.2	8.4
	Perforation: 5	5/5 (100.0)	0/5 (0)	0/5 (0)				13	2
Mennigen 2015	Leak: 45 EVT: 15 SEMS 30	14/15 (93.3)	1/15 (6.6)	0/15 (0)	NA	58	NA	26.5	6.5
		19/30 (63.3)	8/30 (26.6)	0/30 (0)		53		36	1
Loske 2015	Perforation: 10	10/10 (100.0)	0/10 (0)	0/10 (0)	0/10 (0)	NA	NA	5	2
Heits 2014	Perforation: 10	9/10 (90.0)	1/10 (10.0)	NA	NA	48 ^†^	22 ^†^	NA	5.4 ^†^
Liu 2014	Leak: 5	5/5 (100.0)	0/5 (0)	0/5 (0)	NA	NA	NA	34.2	NA
Schorsch 2013	Leak: 17	16/17 (94.1)	1/17 (5.9)	NA	1/17 (5.9)	NA	NA	12	NA
Schniewind 2013	Leak: 29 EVT: 17 SEMS 12	NA	2/17 (11.8)	NA	NA	57 ^†^	26 ^†^	NA	NA
			5/12 (41.7)			62 ^†^	38 ^†^		
Brangewitz 2013	EVT:32	27/32 (84.4)	5/32 (15.6)	9/32 (28.1)	3/32 (9.4)	48.5	NA	23	7
	SEMS: 39	21/39 (53.8)	11/39 (28.2)	3/39 (76.9)	11/39 (28.2)	41		33	3

EVT, endoscopic vacuum therapy; ICU, intensive care unit; NA, not available; SEMS, self-expanding metal stent ^†^ Data expressed as mean. ^‡^ Only total rate was available. ^§^ Two patients died because of fatal hemorrhage during EVT.

## Data Availability

Not applicable.

## Supplementary Materials

The following are available online at https://www.mdpi.com/article/10.3390/jcm10112346/s1: Table S1, search terms in PubMed, Embase and Cochrane Library; Table S2, definition of clinical success, detail indication, and cause of mortality in the 29 studies included; Table S3, detailed information of the 4 studies included patients with fistula; Table S4, patients’ characteristics of studies comparing endoscopic vacuum therapy and self-expanding metal stent, and Table S5, methodological quality.

## References

[1] Freni F., Galletti B., Bruno R., Martines F., Abita P., Gazia F., Sireci F., Galletti F. (2019). Multidisciplinary approach in the removal of post-trauma foreign bodies in the head and neck district: Cases report and review of literature. Acta Med. Mediterr..

[2] Canevari F.R., Martines F., Sorrentino R., Nicolotti M., Sireci F. (2017). Pseudoaneurysm of Superior Thyroid Artery Following A Transesophageal Echocardiography: A Case Presentation. Euromediter. Biomed. J..

[3] Bufkin B.L., Miller J.I., Mansour K.A. (1996). Esophageal perforation: Emphasis on management. Ann. Thorac. Surg..

[4] Altorjay A., Kiss J., Voros A., Bohak A. (1997). Nonoperative management of esophageal perforations. Is it justified?. Ann. Surg..

[5] Brinster C.J., Singhal S., Lee L., Marshall M.B., Kaiser L.R., Kucharczuk J.C. (2004). Evolving options in the management of esophageal perforation. Ann. Thorac. Surg..

[6] Rohatgi A., Papanikitas J., Sutcliffe R., Forshaw M., Mason R. (2009). The role of oesophageal diversion and exclusion in the management of oesophageal perforations. Int. J. Surg..

[7] Kauer W.K., Stein H.J., Dittler H.J., Siewert J.R. (2008). Stent implantation as a treatment option in patients with thoracic anastomotic leaks after esophagectomy. Surg. Endosc..

[8] Huh C.W., Kim J.S., Choi H.H., Lee J.I., Ji J.S., Kim B.W., Choi H. (2018). Treatment of benign perforations and leaks of the esophagus: Factors associated with success after stent placement. Surg. Endosc..

[9] Boulis N.M., Armstrong W.S., Chandler W.F., Orringer M.B. (1999). Epidural abscess: A delayed complication of esophageal stenting for benign stricture. Ann. Thorac. Surg..

[10] Ho H.S., Ong H.S. (2004). A rare life-threatening complication of migrated nitinol self-expanding metallic stent (ultraflex). Surg. Endosc..

[11] Swinnen J., Eisendrath P., Rigaux J., Kahegeshe L., Lemmers A., Le Moine O., Deviere J. (2011). Self-expandable metal stents for the treatment of benign upper GI leaks and perforations. Gastrointest. Endosc..

[12] Bludau M., Fuchs H.F., Herbold T., Maus M.K.H., Alakus H., Popp F., Leers J.M., Bruns C.J., Holscher A.H., Schroder W. (2018). Results of endoscopic vacuum-assisted closure device for treatment of upper gi leaks. Surg. Endosc..

[13] Min Y.W., Kim T., Lee H., Min B.H., Kim H.K., Choi Y.S., Lee J.H., Rhee P.L., Kim J.J., Zo J.I. (2019). Endoscopic vacuum therapy for postoperative esophageal leak. BMC Surg..

[14] Jung C.F.M., Muller-Dornieden A., Gaedcke J., Kunsch S., Gromski M.A., Biggemann L., Hosseini A.S.A., Ghadimi M., Ellenrieder V., Wedi E. (2020). Impact of endoscopic vacuum therapy with low negative pressure for esophageal perforations and postoperative anastomotic esophageal leaks. Digestion.

[15] Wedemeyer J., Schneider A., Manns M.P., Jackobs S. (2008). Endoscopic vacuum-assisted closure of upper intestinal anastomotic leaks. Gastrointest. Endosc..

[16] Loske G., Schorsch T., Muller C. (2010). Endoscopic vacuum sponge therapy for esophageal defects. Surg. Endosc..

[17] Smallwood N.R., Fleshman J.W., Leeds S.G., Burdick J.S. (2016). The use of endoluminal vacuum (E-vac) therapy in the management of upper gastrointestinal leaks and perforations. Surg. Endosc..

[18] Laukoetter M.G., Mennigen R., Neumann P.A., Dhayat S., Horst G., Palmes D., Senninger N., Vowinkel T. (2017). Successful closure of defects in the upper gastrointestinal tract by endoscopic vacuum therapy (EVT): A prospective cohort study. Surg. Endosc..

[19] Virgilio E., Ceci D., Cavallini M. (2018). Surgical endoscopic vacuum-assisted closure therapy (EVAC) in treating anastomotic leakages after major resective surgery of esophageal and gastric cancer. Anticancer Res..

[20] Liberati A., Altman D.G., Tetzlaff J., Mulrow C., Gotzsche P.C., Ioannidis J.P., Clarke M., Devereaux P.J., Kleijnen J., Moher D. (2009). The PRISMA statement for reporting systematic reviews and meta-analyses of studies that evaluate health care interventions: Explanation and elaboration. J. Clin. Epidemiol..

[21] Higgins J.P., Thompson S.G., Deeks J.J., Altman D.G. (2003). Measuring inconsistency in meta-analyses. BMJ.

[22] Brangewitz M., Voigtlander T., Helfritz F.A., Lankisch T.O., Winkler M., Klempnauer J., Manns M.P., Schneider A.S., Wedemeyer J. (2013). Endoscopic closure of esophageal intrathoracic leaks: Stent versus endoscopic vacuum-assisted closure, a retrospective analysis. Endoscopy.

[23] Schniewind B., Schafmayer C., Voehrs G., Egberts J., von Schoenfels W., Rose T., Kurdow R., Arlt A., Ellrichmann M., Jurgensen C. (2013). Endoscopic endoluminal vacuum therapy is superior to other regimens in managing anastomotic leakage after esophagectomy: A comparative retrospective study. Surg. Endosc..

[24] Heits N., Stapel L., Reichert B., Schafmayer C., Schniewind B., Becker T., Hampe J., Egberts J.H. (2014). Endoscopic endoluminal vacuum therapy in esophageal perforation. Ann. Thorac. Surg..

[25] Liu Y.N., Yan Y., Li S.J., Liu H., Wu Q., Zhang L.J., Yang Y., Chen J.F. (2014). Reliable management of post-esophagectomy anastomotic fistula with endoscopic trans-fistula negative pressure drainage. World J. Surg. Oncol..

[26] Schorsch T., Muller C., Loske G. (2014). Endoscopic vacuum therapy of perforations and anastomotic insufficiency of the esophagus. Chirurg.

[27] Loske G., Schorsch T., Dahm C., Martens E., Muller C. (2015). Iatrogenic perforation of esophagus successfully treated with endoscopic vacuum therapy (EVT). Endosc. Int. Open.

[28] Mennigen R., Harting C., Lindner K., Vowinkel T., Rijcken E., Palmes D., Senninger N., Laukoetter M.G. (2015). Comparison of endoscopic vacuum therapy versus stent for anastomotic leak after esophagectomy. J. Gastrointest. Surg..

[29] Moschler O., Nies C., Mueller M.K. (2015). Endoscopic vacuum therapy for esophageal perforations and leakages. Endosc. Int. Open.

[30] Hwang J.J., Jeong Y.S., Park Y.S., Yoon H., Shin C.M., Kim N., Lee D.H. (2016). Comparison of endoscopic vacuum therapy and endoscopic stent implantation with self-expandable metal stent in treating postsurgical gastroesophageal leakage. Medicine.

[31] Kuehn F., Schiffmann L., Janisch F., Schwandner F., Alsfasser G., Gock M., Klar E. (2016). Surgical endoscopic vacuum therapy for defects of the upper gastrointestinal tract. J. Gastrointest. Surg..

[32] Loske G., Schorsch T., Rucktaeschel F., Schulze W., Riefel B., van Ackeren V., Mueller C.T. (2018). Open-pore film drainage (OFD): A new multipurpose tool for endoscopic negative pressure therapy (ENPT). Endosc. Int. Open.

[33] Noh S.M., Ahn J.Y., Lee J.H., Jung H.Y., AlGhamdi Z., Kim H.R., Kim Y.H. (2018). Endoscopic vacuum-assisted closure therapy in patients with anastomotic leakage after esophagectomy: A single-center experience. Gastroenterol. Res. Pr..

[34] Ooi G., Burton P., Packiyanathan A., Loh D., Chen R., Shaw K., Brown W., Nottle P. (2018). Indications and efficacy of endoscopic vacuum-assisted closure therapy for upper gastrointestinal perforations. ANZ J. Surg..

[35] Pournaras D.J., Hardwick R.H., Safranek P.M., Sujendran V., Bennett J., Macaulay G.D., Hindmarsh A. (2018). Endoluminal vacuum therapy (E-vac): A treatment option in oesophagogastric surgery. World J. Surg..

[36] Still S., Mencio M., Ontiveros E., Burdick J., Leeds S.G. (2018). Primary and rescue endoluminal vacuum therapy in the management of esophageal perforations and leaks. Ann. Thorac. Cardiovasc. Surg..

[37] Valli P.V., Mertens J.C., Kroger A., Gubler C., Gutschow C., Schneider P.M., Bauerfeind P. (2018). Stent-over-sponge (SOS): A novel technique complementing endosponge therapy for foregut leaks and perforations. Endoscopy.

[38] Alakkari A., Sood R., Everett S.M., Rembacken B.J., Hayden J., Sarela A., Mohammed N. (2019). First UK experience of endoscopic vacuum therapy for the management of oesophageal perforations and postoperative leaks. Frontline Gastroenterol..

[39] Berlth F., Bludau M., Plum P.S., Herbold T., Christ H., Alakus H., Kleinert R., Bruns C.J., Holscher A.H., Chon S.H. (2019). Self-expanding metal stents versus endoscopic vacuum therapy in anastomotic leak treatment after oncologic gastroesophageal surgery. J. Gastrointest. Surg..

[40] Leeds S.G., Mencio M., Ontiveros E., Ward M.A. (2019). Endoluminal vacuum therapy: How I do it. J. Gastrointest. Surg..

[41] Loske G., Rucktaeschel F., Schorsch T., Moenkemueller K., Mueller C.T. (2019). Endoscopic negative pressure therapy (ENPT) for duodenal leakage—novel repair technique using open-pore film (OFD) and polyurethane-foam drainages (OPD). Endosc. Int. Open.

[42] Morell B., Murray F., Vetter D., Bueter M., Gubler C. (2019). Endoscopic vacuum therapy (EVT) for early infradiaphragmal leakage after bariatric surgery-outcomes of six consecutive cases in a single institution. Langenbecks Arch. Surg..

[43] Rodrigues-Pinto E., Morais R., Vilas-Boas F., Pereira P., Macedo G. (2019). Role of endoscopic vacuum therapy, internal drainage, and stents for postbariatric leaks. VideoGIE.

[44] Walsh L.T., Loloi J., Manzo C.E., Mathew A., Maranki J., Dye C.E., Levenick J.M., Taylor M.D., Moyer M.T. (2019). Successful treatment of large cavity esophageal disruptions with transluminal washout and endoscopic vacuum therapy: A report of two cases. Adv. Gastrointest. Endosc..

[45] Watson A., Zuchelli T. (2019). Repair of upper-GI fistulas and anastomotic leakage by the use of endoluminal vacuum-assisted closure. VideoGIE.

[46] Jeon J.H., Jang H.J., Han J.E., Park Y.S., Seong Y.W., Cho S., Jheon S., Kim K. (2020). Endoscopic vacuum therapy in the management of postoperative leakage after esophagectomy. World J. Surg..

[47] Palmes D., Kebschull L., Bahde R., Senninger N., Pascher A., Laukotter M.G., Eichelmann A.K. (2020). Management of nonmalignant tracheo- and bronchoesophageal fistula after esophagectomy. Thorac. Cardiovasc. Surg..

[48] Kuehn F., Loske G., Schiffmann L., Gock M., Klar E. (2017). Endoscopic vacuum therapy for various defects of the upper gastrointestinal tract. Surg. Endosc..

[49] Verstegen M.H.P., Bouwense S.A.W., van Workum F., Ten Broek R., Siersema P.D., Rovers M., Rosman C. (2019). Management of intrathoracic and cervical anastomotic leakage after esophagectomy for esophageal cancer: A systematic review. World J. Emerg. Surg..

[50] Newton N.J., Sharrock A., Rickard R., Mughal M. (2017). Systematic review of the use of endo-luminal topical negative pressure in oesophageal leaks and perforations. Dis. Esophagus.

[51] Argenta L.C., Morykwas M.J. (1997). Vacuum-assisted closure: A new method for wound control and treatment: Clinical experience. Ann. Plast. Surg..

[52] Wedemeyer J., Brangewitz M., Kubicka S., Jackobs S., Winkler M., Neipp M., Klempnauer J., Manns M.P., Schneider A.S. (2010). Management of major postsurgical gastroesophageal intrathoracic leaks with an endoscopic vacuum-assisted closure system. Gastrointest. Endosc..

[53] Schaheen L., Blackmon S.H., Nason K.S. (2014). Optimal approach to the management of intrathoracic esophageal leak following esophagectomy: A systematic review. Am. J. Surg..

