# Supplementary material for: Endoscopic Vacuum Therapy in Patients with Transmural Defects of the Upper Gastrointestinal Tract: A Systematic Review with Meta-Analysis
Source: J Clin Med. 2021 May 27;10(11):2346. doi: 10.3390/jcm10112346 (PMC8197794; doi:10.3390/jcm10112346)
Supplement: Supplementary file 1 [file jcm-10-02346-s001.zip › jcm-1205490-supplementary.pdf]

Supplementary Table S1. Search terms in PubMed, Embase and Cochrane Library

PubMed	Embase	Cochrane Library
((Anastomotic leak OR anastomotic leakage OR postoperative leak OR postoperative leakage OR esophageal leak OR esophageal leakage OR esophageal fistula OR leakage OR fistula OR leak OR perforation OR upper gastrointestinal tract OR esophagus OR esophageal OR gastric OR stomach OR esophagectomy OR anastomosis)) OR ((("Anastomotic Leak"[Mesh]) OR "Esophageal Fistula"[Mesh]) OR "Fistula"[Mesh]) OR "Upper Gastrointestinal Tract"[Mesh]) OR "Esophagus"[Mesh]) OR "Stomach"[Mesh]) OR "Esophagectomy"[Mesh]) OR "Anastomosis, Surgical"[Mesh]))) AND (((endoscopic vacuum therapy OR endoscopic vacuum assisted closure OR endoluminal vacuum therapy OR vacuum therapy OR vacuum assisted closure OR negative pressure wound therapy OR endoscopic negative pressure therapy OR negative pressure therapy OR endovac therapy OR endo sponge)) OR "Negative-Pressure Wound Therapy"[Mesh]))) AND (((treatment outcome OR mortality OR hospital duration OR treatment duration OR complication)) OR ("Treatment Outcome"[Mesh] OR "Mortality"[Mesh] OR "Duration of Therapy"[Mesh])))	(('anastomotic leak' OR 'anastomotic leakage' OR 'postoperative leak' OR 'postoperative leakage' OR 'esophageal leak' OR 'esophageal leakage' OR 'esophageal fistula' OR leakage OR fistula OR leak OR perforation OR 'upper gastrointestinal tract' OR esophagus OR esophageal OR gastric OR stomach OR esophagectomy OR anastomosis) OR ('anastomosis leakage'/exp OR 'postoperative leak'/exp OR 'esophageal leak'/exp OR 'esophagus fistula'/exp OR 'leakage'/exp OR 'fistula'/exp OR 'perforation'/exp OR 'upper gastrointestinal tract'/exp OR 'esophagus'/exp OR 'stomach'/exp)) AND (('endoscopic vacuum therapy' OR 'endoscopic vacuum assisted closure' OR 'endoluminal vacuum therapy' OR 'vacuum therapy' OR 'vacuum assisted closure' OR 'negative pressure wound therapy' OR 'endoscopic negative pressure therapy' OR 'negative pressure therapy' OR 'endovac therapy' OR 'endo sponge') OR ('endoscopic vacuum therapy'/exp OR 'endoluminal vacuum therapy'/exp OR 'vacuum assisted closure'/exp OR 'vacuum therapy device'/exp)) AND (('treatment outcome' OR mortality OR 'hospital duration' OR 'treatment duration' OR complication) OR ('treatment outcome'/exp OR 'mortality'/exp OR 'treatment duration'/exp OR 'complication'/exp))	((anastomotic leak OR anastomotic leakage OR postoperative leak OR postoperative leakage OR esophageal leak OR esophageal leakage OR esophageal fistula OR leakage OR fistula OR leak OR perforation OR upper gastrointestinal tract OR esophagus OR esophageal OR gastric OR stomach OR esophagectomy OR anastomosis) OR ([Anastomotic Leak] explode all trees) OR ([Esophageal Fistula] explode all trees) OR ([Fistula] explode all trees) OR ([Upper Gastrointestinal Tract] explode all trees) OR ([Esophagus] explode all trees) OR ([Stomach] explode all trees) OR ([Esophagectomy] explode all trees) OR ([Anastomosis, Surgical] explode all trees)) AND ((endoscopic vacuum therapy OR endoscopic vacuum assisted closure OR endoluminal vacuum therapy OR vacuum therapy OR vacuum assisted closure OR negative pressure wound therapy OR endoscopic negative pressure therapy OR negative pressure therapy OR endovac therapy OR endo sponge) OR ([Negative-Pressure Wound Therapy] explode all trees)) AND (treatment outcome OR mortality OR hospital duration OR treatment duration OR complication) OR ([Treatment Outcome] explode all trees) OR ([Mortality] explode all trees) OR ([Duration of Therapy] explode all trees)

Supplementary Table S2. Definition of clinical success, detail indication, and cause of mortality in the 29 studies included

Authors	Definition of clinical success	Detail indication	Mortality (n)	Cause of mortality
**Palmes 2020**	NA	Post-op fistula(n=4): 4 Ivor Lewis (29% SCC, 71% EAC)	2	sepsis due to persistent leak (closure fail)
**Jung 2019**	closure of defect under endoscopy	Post-op leak(n=23): 15 Ivor Lewis, 8 gastrectomy (5 SCC, 17 EAC, 1 NET)	1	multi-organ failure after mixed cardiogenic and septic shock (closure fail)
		Perforation(n=7): 2 endoscopic dilation, 1 TEE, 1 postoperative torquation, 1 endoscopy, 1 Boerhaave, 1 ischemia	1	underlying advanced esophageal cancer (closure fail)
**Jeon 2019**	NA	Post-op leak(n=22): 14 Ivor Lewis, 4 modified McKeown, 2 colon interposition, 2 gastrectomy (19 SCC, 2 EAC, 1 GIST)	0	-
**Watson 2019**	closure of defect under endoscopy and esophagogram	Post-op leak(n=2): 2 Ivor Lewis (2 EAC)<br>Post-op fistula(n=1): gastrectomy	0	-
**Pinto 2019**	NA	Post-op leak(n=2): 1 RYGB, 1 sleeve gastrectomy	0	-
**Morell 2019**	closure of defect under endoscopy	Post-op leak(n=6): 4 RYGB, 2 sleeve gastrectomy	0	-
**Min 2019**	closure of defect under endoscopy	Post-op leak(n=20): 7 Ivor Lewis, 8 3-field lymph node dissection, 4 McKeown, 1 Transhiatal esophagectomy	1	sepsis due to persistent leak (closure fail)
**Loske 2019**	closure of defect under endoscopy	Post-op leak(n=1): 1 Billroth-1 gastro-jejunostomy	0	
		Perforation(n=10): 1 endoscopic dilation, 1 operative drain, 1 surgical papillectomy, 1 ERCP, 6 perforated ulcer	2	NA (closure success)
**Leeds 2019**	closure of defect under endoscopy or esophagogram	NA	NA	NA
**Walsh 2019**	closure of defect under endoscopy and esophagogram	Post-op leak(n=1): 1 Ivor Lewis	1	aspiration pneumonia (closure success)
		Perforation(n=1): 1 Boerhaave	0	
**Alakkari 2019**	closure of defect under esophagogram	Post-op leak(n=1): 1 Merendino esophageal reconstruction	0	-
		Perforation(n=1): 1 spontaneous esophageal perforation	0	-
**Berlth 2018**	closure of defect under endoscopy	Post-op leak(n=111)<br>EVT(n=34): 74% Ivor Lewis<br>SEMS(n=77): 88% Ivor Lewis	14<br>3<br>11	11 sepsis due to persistent leak(closure fail), 3 sepsis due to other cause (closure success)
**Valli 2018**	closure of defect under endoscopy or esophagogram	Post-op leak(n=11): 6 esophagogastrostomy, 3 esophagojejunostomy, 1 gastrojejunostomy, 1 sleeve gastrectomy	0	
		Fistula(n=1): 1 TEE	1	persistent leak (closure fail)
**Still 2018**	closure of defect under endoscopy or esophagogram	Post-op leak(n=2): anastomosis site leak<br>Perforation(n=9): 8 iatrogenic perforation, 1 Boerhaave,<br>Post-op leak(n=2): 2 bronchoesophageal fistula	NA	NA
**Pournaras 2018**	closure of defect under endoscopy	Post-op leak(n=14): anastomosis site leak	0	
		Perforation(n=7): 7 iatrogenic perforation	1	withdrawing consent for further intervention following one EVT

**Ooi 2018**	closure of defect under endoscopy or esophagogram	Post-op leak(n=6): 2 esophagectomy, 2 sleeve gastrectomy, 1 total gastrectomy, 1 intra-op injury due to gastric band	1	sepsis due to persistent leak (closure fail)
		Perforation(n=4): 2 endoscopic dilation, 1 Boerhaave, 1 TEE	1	sepsis due to persistent leak (closure fail)
**Noh 2018**	closure of defect under endoscopy and esophagogram	Post-op leak(n=12): 11 Ivor Lewis, 1 esophageal diverticulectomy	1	aspiration pneumonia (closure success)
**Loske 2018**	NA	Post-op leak(n=3): 2 Ivor Lewis, 1 gastrectomy	0	-
		Perforation(n=1): 1 Boerhaave	0	-
**Laukoetter 2017**	closure of defect under endoscopy	Post-op leak(n=39): anastomosis site leak	5	1 multiorgan failure due to persistent leak (closure fail), 2 EVT related fatal bleeding (closure success), 1 pneumonia (closure success)
		Perforation(n=13): 9 iatrogenic perforation, 4 Boerhaave	0	
**Kuehn 2016**	NA	Post-op leak(n=11): anastomosis site leak	1	sepsis due to persistent leak (closure fail)
		Perforation(n=10): 8 iatrogenic perforation, 2 Boerhaave	0	
**Hwang 2016**	closure of defect under endoscopy or esophagogram	Post-op leak(n=18)		
		EVT(n=7): 71% Ivor Lewis	0	
		SEMS(n=11): 36% Ivor Lewis	2	cancer progression (closure fail)
**Möschler 2015**	NA	Post-op leak(n=5): 5 thoracoabdominal resection of the esophagus	2	cardiovascular disease (closure success)
		Perforation(n=5): 3 iatrogenic perforation, 2 foreign body	0	
**Mennigen 2015**	closure of defect under endoscopy and esophagogram	Post-op leak(n=45)		
		EVT(n=15): 100% Ivor Lewis	1	pneumonia (closure success)
		SEMS(n=30): 100% Ivor Lewis	8	4 sepsis (closure success), 4 sepsis (closure fail)
**Loske 2015**	NA	Perforation(n=10): 8 iatrogenic perforation, 2 foreign body	0	-
**Heits 2014**	closure of defect under endoscopy	Perforation(n=10): 4 iatrogenic perforation, 1 foreign body, 5 Boerhaave	1	Heart failure (closure success)
**Liu 2014**	NA	Post-op leak(n=5): 4 Ivor Lewis, 1 McKeown	0	-
**Schorsch 2013**	NA	Post-op leak(n=17): 7 thoracoabdominal esophagogastrectomy, 5 transhiatal esophagogastrectomy, 5 gastrectomy	1	1 sepsis (closure fail)
**Schniewind 2013**	closure of defect under endoscopy or esophagogram	Post-op leak(n=29)		
		EVT(n=17): 100% Ivor Lewis	2	NA
		SEMS(n=12): 100% Ivor Lewis	5	NA
**Brangewitz 2013**	closure of defect under endoscopy or esophagogram	EVT(n=32): 94% anastomosis site leak	5	NA
		SEMS(n=39): 79% anastomosis site leak	11	NA

EAC esophageal adenocarcinomal; ERCP, endoscopic retrograde choangiopancreatography; EVT, endoscopic vacuum therapy; NA, not available; NET, neuroendocrine tumor; RYGB, Roux-en-Y gastric bypass; SEMS, self-expanding metal stent; SCC, squamous cell carcinoma; TEE transesophageal echocardiography

Supplementary Table S3. Detail information of the 4 studies included patients with fistula

Authors	Definition of fistula	Detail information	Outcome
**Palmes 2020**	Tracheoesophageal fistula (TEF) or bronchoesophageal fistula (BEF)	2 Lt.BEF, 1 Rt.BEF, 1 TEF	2 Clinical success<br>2 Clinical fail → sepsis → death
**Watson 2019**	NA	1 gastroesophageal junction fistula	1 Clinical success
**Valli 2018**	NA	Chronic fistula after TEE	1 Clinical fail → sepsis → death
**Still 2018**	NA	2 BEF (EVT + bronchial stent placement)	NA

EVT, endoscopic vacuum therapy; NA, not available; TEE transesophageal echocardiography

Supplementary Table S4. Patients characteristics of studies comparing endoscopic vacuum therapy and self-expanding metal stent

Study, treatment method, n	Patients, n	Male, n	Age (median, years)	Negative pressure (mmHg)	Oncologic resection, %	Neoadjuvant therapy, %	Etiology :surgical anastomotic leak, %	(Sub-) total esophagectomy, %	Reconstruction type esophagogastrostomy, %	Type of stent	Defect size (median, mm)
**Brangewitz 2013**											
EVT	32	28	63 (45–84)	125	88	56	94	NA	44	NA	NA
SEMS	39	30	62 (32–78)	NA	74	15	79	NA	69	Fully covered	NA
**Schniewind 2013**											
EVT	17	NA	NA	70-80	NA	NA	100	100	NA	NA	NA
SEMS	12	NA	NA	NA	NA	NA	100	100	NA	NA	NA
**Mennigen 2015**											
EVT	15	14	56 (42-76)	100-125	100	73	100	100	100	NA	NA
SEMS	30	21	66 (40-92)	NA	93	43	100	100	100	Fully covered	NA
**Hwang 2016**											
EVT	7	5	71 (63-78)	125	100	NA	100	71	NA	NA	8.1
SEMS	11	9	67 (55-81)	NA	100	NA	100	36	NA	fully or partially covered	6.6
**Berlth 2018**											
EVT	34	29	65 (43-84)	125	100	53	100	74	74	NA	NA
SEMS	77	63	64 (43–88)	NA	100	65	100	88	88	Fully covered	NA

EVT, endoscopic vacuum therapy; NA, not available; SEMS, self-expanding metal stent

Supplementary Table S5. Methodological quality

Authors	Selection Representativeness	Selection Selection	Selection Ascertainment	Selection Outcome of interest	Comparability Comparability	Outcome Assessment of outcome	Outcome Duration of follow-up	Outcome Adequacy of follow-up	Total Score (maximum: 9 stars)
Palmes 2020	0	NA	*	*	NA	*	NA	NA	3
Jung 2019	*	NA	*	*	NA	*	*	0	5
Jeon 2019	*	NA	*	*	NA	*	*	0	5
Watson 2019	0	NA	*	*	NA	*	0	0	3
Pinto 2019	0	NA	*	*	NA	*	0	0	3
Morell 2019	0	NA	*	*	NA	*	0	0	3
Min 2019	*	NA	*	*	NA	*	*	0	5
Loske 2019	0	NA	*	*	NA	*	0	0	3
Leeds 2019	*	NA	*	*	NA	*	0	0	4
Walsh 2019	0	NA	*	*	NA	*	0	0	3
Alakkari 2019	0	NA	*	*	NA	*	0	0	3
Berlth 2018	*	NA	*	*	0	*	0	0	4
Valli 2018	0	NA	*	*	NA	*	0	0	3
Still 2018	0	NA	*	*	NA	*	0	0	3
Pournaras 2018	*	NA	*	*	NA	*	0	0	4
Ooi 2018	0	NA	*	*	NA	*	0	0	3
Noh 2018	*	NA	*	*	NA	*	*	*	6
Loske 2018	0	NA	*	*	NA	*	0	0	3
Laukoetter	*	NA	*	*	NA	*	*	*	6
Kuehn 2016	*	NA	*	*	NA	*	*	*	6
Hwang 2016	*	NA	*	*	0	*	0	0	4
Möschler 2015	0	NA	*	*	NA	*	0	0	3
Mennigen 2015	*	NA	*	*	0	*	0	0	4
Loske 2015	0	NA	*	*	NA	*	0	0	3
Heits 2014	0	NA	*	*	NA	*	0	0	3
Liu 2014	0	NA	*	*	NA	*	0	0	3
Schorsch 2013	*	NA	*	*	NA	*	0	0	4
Schniewind	*	NA	*	0	*	*	0	0	4
Brangewitz	*	NA	*	*	0	*	0	0	4
